# Treatment for intermediate and advanced-stage hepatocellular carcinoma: does systemic therapy synergize the therapeutic efficacy of TACE?

**DOI:** 10.3389/fimmu.2026.1819885

**Published:** 2026-05-01

**Authors:** Zhuo Li, Jie Tan, Zhi-Jun Li, Peng Yan, Hua Xiang

**Affiliations:** 1 Department of Interventional Vascular Surgery, Hunan Provincial People’s Hospital (The First Affiliated Hospital of Hunan Normal University), Changsha, China; 2 Department of Interventional Vascular Surgery, Hunan Cancer Hospital, The Affiliated Cancer Hospital of Xiangya School of Medicine, Central South University, Changsha, Hunan, China

**Keywords:** hepatocellular carcinoma, overall survival, patient selection, systemic therapy, transarterial chemoembolization

## Abstract

**Background:**

Transarterial chemoembolization (TACE) is a standard treatment for intermediate-stage hepatocellular carcinoma (HCC), but its efficacy varies due to patient heterogeneity. Combining TACE with systemic therapies, such as targeted agents or immune checkpoint inhibitors, has shown promise in improving outcomes, though controversies regarding survival benefits and safety persist. This study investigates whether synchronized systemic therapy enhances the therapeutic efficacy of TACE in intermediate and advanced-stage HCC.

**Methods:**

This single-center, retrospective study included 142 patients with intermediate and advanced-stage HCC (BCLC B or C) who received TACE as initial treatment between February 2019 and August 2022 at Hunan Provincial People’s Hospital. Patients were divided into two groups: combination therapy (TACE plus systemic therapy, n=41) and TACE monotherapy (n=101). Progression-free survival (PFS), overall survival (OS), treatment response (per mRECIST criteria), and adverse events (AEs) were compared. Cox regression and Kaplan-Meier analyses were used to assess survival outcomes.

**Results:**

No significant differences were observed in baseline characteristics, except for a higher proportion of Child-Pugh A patients in the combination group (90.2% vs. 67.3%, P= 0.005). Median PFS was similar between the combination and monotherapy groups (5.5 vs. 6.0 months, P= 0.832), with no significant differences in BCLC-B (18.3 vs. 17.4 months, P= 0.516) or BCLC-C (4.1 vs. 3.7 months, P= 0.255) subgroups. However, the combination group showed a trend toward improved OS (24.8 vs. 16.7 months, P= 0.282), with a significant benefit in BCLC-C patients (17.9 vs. 11.0 months, P= 0.048). Grade 3 or 4 AEs were comparable between groups (7.2% vs. 14.9%, P= 0.187).

**Conclusion:**

Combining systemic therapy with TACE does not improve PFS but may enhance OS in BCLC-C patients. The safety profile is comparable, particularly in patients with preserved liver function. These findings highlight the importance of patient selection and warrant further prospective studies to optimize treatment strategies.

## Introduction

Transarterial chemoembolization (TACE) is a cornerstone treatment for intermediate-stage hepatocellular carcinoma (HCC), recommended for its ability to control tumor burden and extend survival. However, the heterogeneity of BCLC B patients—encompassing tumor size, number, alpha-fetoprotein (AFP) levels, and liver function—limits the efficacy of TACE monotherapy in certain subgroups, prompting exploration of TACE combined with systemic therapies, particularly targeted therapies and immune checkpoint inhibitors (collectively termed “systemic therapy”). Recent studies have demonstrated the potential of these combinations to improve outcomes. Specifically, the CHANCE001 and CHANCE2211 studies reported significant improvements in progression-free survival (PFS, 9.5–13.5 months vs. 7.7–8.0 months), overall survival (OS, 19.2–24.1 months vs. 15.7 months), and objective response rate (ORR, 59.5%–60.1% vs. 32.0%–37.4%) with TACE plus targeted-immunotherapy compared to TACE alone (1, 2). The LAUNCH trial demonstrated a significant improvement in PFS (10.6 months vs. 6.4 months) with TACE plus Lenvatinib in advanced-stage HCC patients, while the TACTICS-L trial, targeting intermediate-stage (BCLC B) HCC, showed an extended PFS of up to 28.0 months using a similar combination strategy (3, 4).

Despite these advances, the application of TACE combined with systemic therapy remains controversial. Several trials, including SPACE and TACE-2, found no significant OS benefit from combining TACE with sorafenib, with increased severe adverse events such as diarrhea, abdominal pain, fatigue, and hand-foot skin reactions. Notably, these trials included predominantly Child-Pugh A patients without high-risk features such as portal vein tumor thrombosis (PVTT) (5, 6). Additionally, patient selection remains a challenge, as those with single, small tumors may achieve sufficient control with TACE or locoregional therapies alone. In contrast, high-risk patients may benefit more from systemic therapies like atezolizumab plus bevacizumab (7). Furthermore, the high cost and cumulative toxicity of combined regimens limit their widespread adoption, particularly in resource-constrained settings (8). Thus, while TACE combined with systemic therapy holds promise for synergistic effects in BCLC B HCC, controversies regarding its optimal indications, patient selection, and cost-effectiveness necessitate further research to refine individualized treatment strategies.

Based on the controversial background, we conducted a retrospective comparative study to investigate whether synchronized systemic therapy could synergize the efficacy of TACE for intermediate and advanced HCC.

## Materials and methods

### Study design and participants

**Figure 1:**
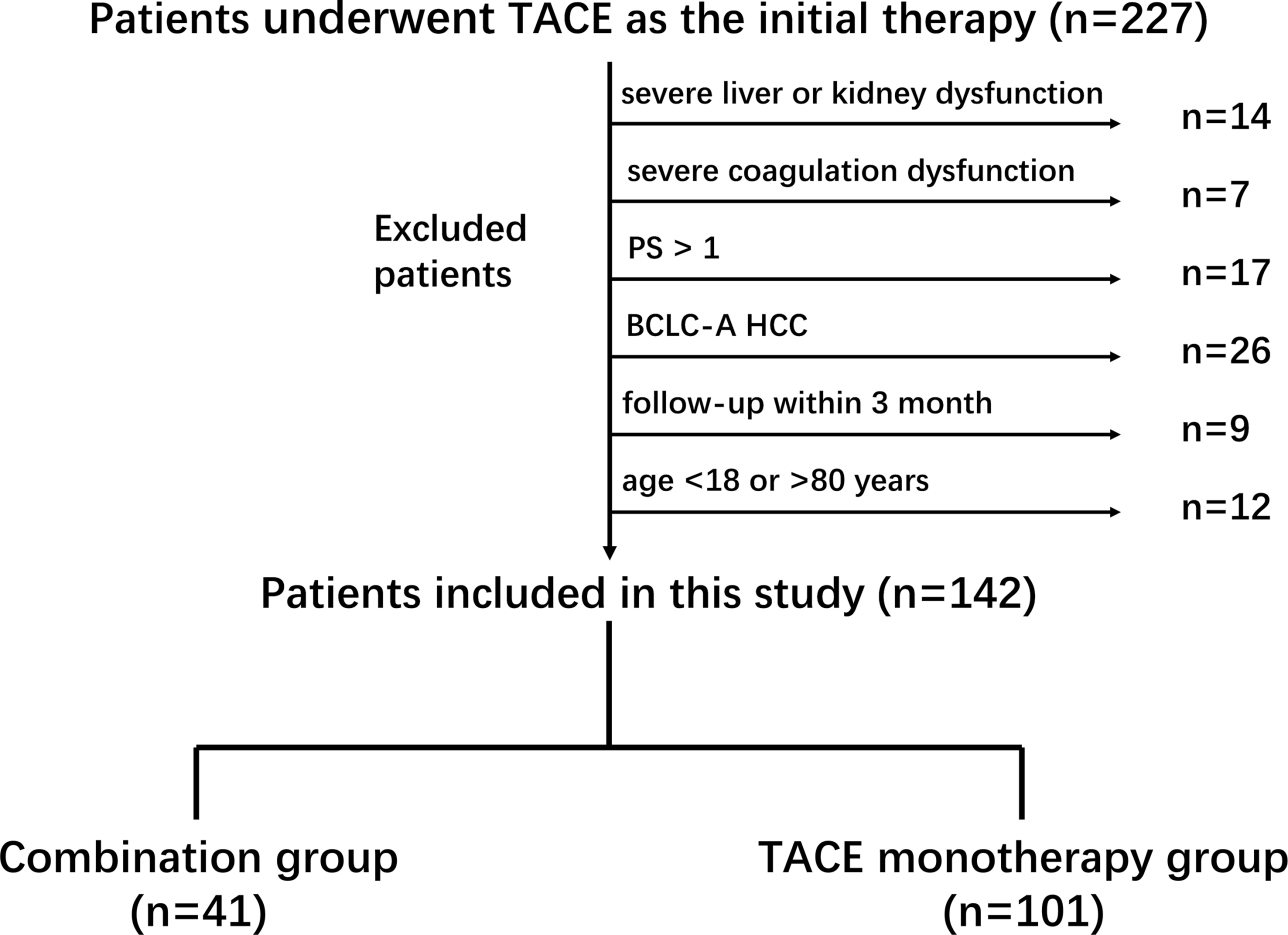
Flowchart of patient selection and inclusion process.

This was a retrospective, single-center study approved by the Ethics Committee of Hunan Provincial People’s Hospital (First Affiliated Hospital of Hunan Normal University) and conducted in accordance with the Declaration of Helsinki. The requirement for informed consent was waived due to the retrospective nature. A total of 227 patients diagnosed with HCC and treated with TACE as the initial therapy between February 2019 and August 2022 were retrospectively screened. Diagnosis was confirmed using either the Liver Imaging Reporting and Data System (LI-RADS) criteria or histopathological evaluation (9). Exclusion criteria included severe hepatic or renal dysfunction, uncorrectable coagulopathy, ECOG performance status >1, BCLC stage A, follow-up <3 months, and age <18 or >80 years. After applying these criteria, 142 intermediate and advanced-stage HCC patients were included in the analysis (**Figure 1**).

### Data collection and definition

Demographic and clinical variables, including age, sex, Barcelona Clinic Liver Cancer (BCLC) stage, tumor rupture status, Child–Pugh classification, alanine transaminase (ALT), aspartate aminotransferase (AST), alkaline phosphatase (ALP), complete blood counts (neutrophils, lymphocytes, monocytes, platelets), hepatitis etiology, alpha-fetoprotein (AFP) levels, type of initial TACE, treatment response to initial TACE, and whether systemic therapy combined, were extracted from the electronic medical records. Inflammatory markers were computed using peripheral blood counts as follows: NLR = neutrophil count/lymphocyte count; LMR = lymphocyte count/monocyte count; PLR = platelet count/lymphocyte count. Patients were categorized based on treatment type into a combination group (TACE plus systemic therapy) or a TACE monotherapy group.

### TACE procedure and systemic therapy

TACE was indicated for HCC patients ineligible for or unwilling to undergo curative treatments such as surgery or transplantation. All procedures were performed by experienced interventional radiologists using femoral or radial artery access. For femoral artery access, 5-Fr Yashiro or Rosch hepatic catheters were used, while vertebral or multipurpose catheters were employed for radial artery access. Angiographic evaluation was used to localize tumor-feeding vessels, followed by superselective catheterization using microcatheters.

In conventional TACE (cTACE), an emulsion was prepared using up to 15 mL of iodized oil (Lipiodol) mixed with chemotherapeutic agents—either 10 mg idarubicin or 40–80 mg doxorubicin—at a 3:1 or 2:1 ratio of oil to aqueous solvent (sugar solution or sterile water) (10). The endpoint of Lipiodol injection is portal vein visualization adjacent to the tumor (grade 1) (11). Tumor feeding artery embolization was then performed using Gelfoam slurry, microspheres (Embozene, Varian Medical Systems), or polyvinyl alcohol (PVA) particles (Cook Medical). For patients with hepatic arteriovenous fistulas (AVF), embolic agents such as PVA or microspheres were delivered first to minimize the risk of nontarget embolization. In drug-eluting bead TACE (DEB-TACE), the same chemotherapeutic agents were loaded into beads (Biocompatibles, UK; Jiangsu Hengrui, China) for 20–30 minutes before being selectively infused into tumor-feeding arteries under angiographic guidance, enabling sustained local drug release and prolonged tumor exposure. For HCC lesions without AVF during superselective angiography, drug-eluting beads with a diameter of less than 300 μm were preferentially selected to enhance tumor penetration and maximize embolic efficacy. All procedures adhered to the Society of Interventional Radiology (SIR) guidelines, ensuring standardized, evidence-based practice to maximize safety and efficacy (12). For patients with HCC lesions distributed in both lobes, schedule TACE sessions can be performed (13). Post-procedure adverse events (AEs) were documented according to the Common Terminology Criteria for Adverse Events (CTCAE) (14).

In the combination group, systemic therapy consisted of a combination of first-line targeted agents and immune checkpoint inhibitors (ICIs). Targeted therapies included Sorafenib, Lenvatinib, Donafenib, or Bevacizumab, administered at standard doses. Immunotherapy involved the administration of PD-1 inhibitors, specifically Sintilimab or Tislelizumab (200 mg intravenously every 3 weeks). Systemic therapy was initiated within 3–5 days following the first TACE session, provided the patient’s liver function had sufficiently recovered to tolerate subsequent systemic therapy.

### Follow-up and outcome

Following the initial TACE, patients were monitored through scheduled imaging evaluations, including contrast-enhanced liver CT or MRI and chest CT, to assess treatment response and guide further management. Additional TACE sessions were administered based on radiologic evidence of residual viable tumor and hepatic functional reserve. Tumor response was evaluated using the modified Response Evaluation Criteria in Solid Tumors (mRECIST), incorporating assessments of target lesions, non-target lesions, and new disease foci. Based on mRECIST, responses were categorized as complete response (CR), partial response (PR), stable disease (SD), or progressive disease (PD) (15). CR and PR were considered indicative of effective treatment, while SD and PD were classified as ineffective responses (16). The primary endpoint was progression-free survival (PFS), defined as the time from the initial TACE to the first documentation of disease progression, death, or the last follow-up.

The primary outcome was progression-free survival (PFS), defined as the interval from the first TACE to either radiologic progression, death, or the last documented follow-up. Overall survival (OS) served as the secondary endpoint, measured from the date of initial TACE to death from any cause or the last follow-up. Follow-up concluded in June 2025, with durations ranging from 3.4 to 68 months (median: 16.5 months). Patients without events at the time of last contact were censored accordingly.

### Statistical methods

Statistical analyses were conducted using SPSS version 27.0 (IBM Corp.) and R software version 4.0.2 (http://www.R-project.org). The distribution of continuous variables was assessed using the Kolmogorov–Smirnov test. Variables with normal distribution were summarized as mean ± standard deviation (SD), while non-normally distributed data were presented as median with interquartile range (IQR). Between-group comparisons were performed using the independent t-test or Mann–Whitney U test, depending on data distribution. Categorical variables were reported as frequencies and percentages, with group differences analyzed via the χ² test or Fisher’s exact test, as appropriate. Survival outcomes, including PFS and OS, were estimated using the Kaplan–Meier method and compared with the log-rank test. Cox proportional hazards regression was employed to identify independent prognostic factors. Variables with a univariate *P*-value < 0.1 were entered into multivariate models. Survival analysis was performed using the R packages survival and survminer (17, 18). A two-tailed *P*-value < 0.05 was considered statistically significant. As this was a retrospective study, an *a priori* sample size calculation was not performed. Instead, all eligible patients during the study period were included to maximize statistical power. A *post-hoc* power analysis was conducted to verify the reliability of the observed significant differences in survival outcomes.

## Results

### Clinical baseline characteristics

A total of 142 patients were included, comprising 41 in the combination therapy group and 101 in the TACE monotherapy group. Baseline characteristics such as gender, age, incidence of HCC rupture, hepatitis status, alpha-fetoprotein (AFP) levels, inflammatory indices (NLR, LMR, PLR, and SII), and TACE modality showed no significant differences between the two groups. However, the proportion of patients with Child-Pugh class A was significantly higher in the combination group than in the monotherapy group (90.2% vs. 67.3%, *P*= 0.005), indicating better baseline liver function. Although the difference was not statistically significant, the combination group exhibited a higher rate of effective TACE response (75.6% vs. 61.4%, *P*= 0.106). Detailed statistics are summarized in **Table 1**.

**Table 1:** The baseline characteristics of all patients included in analysis.

Characteristics	Combination group (n=41) (%)	TACE monotherapy group (n=101) (%)	P value
Gender			0.704
Male	33 (80.5)	84 (83.2)	
Female	8 (19.5)	17 (16.8)	
Age			0.068
<60	26 (63.4)	47 (46.5)	
≥60	15 (36.6)	54 (53.5)	
BCLC stage			0.129
Stage B	13 (31.7)	46 (45.5)	
Stage C	28 (68.3)	55 (54.5)	
HCC lesion rupture			0.129
Yes	6 (14.6)	14 (13.9)	
No	35 (85.4)	87 (86.1)	
Child-Pugh grade			0.005
A	37 (90.2)	68 (67.3)	
B	4 (9.8)	33 (32.7)	
Hepatitis			0.836
None	3 (7.3)	8 (7.9)	
HBV	29 (70.3)	75 (74.3)	
HCV	1 (2.4)	4 (4.0)	
Alcoholic	8 (19.5)	14 (13.9)	
AFP (ng/mL)			0.819
<400	24 (58.5)	57 (56.4)	
≥400	17 (41.5)	44 (43.6)	
NLR	3.12 (2.15-4.37)	2.97 (1.82-4.69)	0.709
LMR	2.52 (1.64-3.68)	2.45 (1.90-3.21)	0.695
PLR	123.91 (95.26-184.17)	111.54 (78.97-184.98)	0.471
TACE Type			0.430
c-TACE	8 (19.5)	26 (25.7)	
DEB-TACE	33 (80.5)	75 (74.3)	
TACE overall response			0.106
Effective	31 (75.6)	62 (61.4)	
Ineffective	10 (24.4)	39 (38.6)	

TACE, Transcatheter Arterial Chemoembolization; BCLC, Barcelona Clinic Liver Cancer; HCC: Hepatocellular Carcinoma; HBV, Hepatitis B Virus; HCV, Hepatitis C Virus; AFP, Alpha-fetoprotein; NLR, Neutrophil to Lymphocyte Ratio; LMR, Lymphocyte to Monocyte Ratio; PLR, Platelet to Lymphocyte Ratio; c-TACE, conventional TACE; DEB-TACE, Drug Eluting Beads TACE.

### Cox regression analysis for PFS and OS

Univariate analysis identified BCLC stage, AFP level, PLR, TACE modality, and treatment response as significant predictors of progression-free survival (PFS). Multivariate analysis demonstrated that BCLC stage C (HR= 2.484, 95% CI: 1.455–4.242, *P*= 0.001) and an ineffective TACE response (HR= 1.893, 95% CI: 1.208–2.967, *P*= 0.005) were independently associated with shorter PFS. Detailed results are presented in **Table 2**.

**Table 2:** Uni- and multivariate Cox regression analysis of the risk factors associated with PFS.

	Univariate analysis		Multivariate analysis	
Variable	HR (95% CI)	P-value	HR (95% CI)	P-value
Gender		0.503	–	–
Male	Reference			
Female	1.184 (0.723, 1.939)			
Age		0.793		
<60	Reference			
≥60	1.504 (0.710, 1.567)			
BCLC stage		<0.001		0.001
Stage B	Reference		Reference	
Stage C	3.041 (1.977, 4.679)		2.484 (1.455, 4.242)	
HCC lesion rupture		0.310		
Yes	Reference			
No	0.757 (0.434, 1.319)			
Child-Pugh grade		0.458		
A	Reference			
B	1.192 (0.754, 1.883)			
Hepatitis		0.287		
None	Reference			
HBV	1.257 (0.603, 2.691)			
HCV	0.195 (0.024, 1.605)			
Alcoholic	1.311 (0.555, 3.099)			
AFP (ng/mL)		<0.001		0.139
<400	Reference		Reference	
≥400	2.597 (1.733, 3.891)		1.449 (0.887, 2.369)	
NLR	1.051 (0.997, 1.107)	0.062	1.063 (0.962, 1.174)	0.229
LMR	0.909 (0.801, 1.031)	0.137		
PLR	1.002 (1.001, 1.004)	0.011	0.998 (0.995–1.001)	0.218
TACE Type		0.025		0.128
c-TACE	Reference		Reference	
DEB-TACE	1.762 (1.074, 2.889)		1.487 (0.892, 2.481)	
Initial therapy modality		0.833		
Combination	Reference			
TACE monotherapy	0.956 (0.630, 1.450)			
TACE overall response		<0.001		0.005
Effective	Reference		Reference	
Ineffective	2.228 (1.492–3.326)		1.893 (1.208, 2.967)	

PFS, Progression Free Survival; HR: Hazard Ratio; 95% CI: 95% Confidence Interval; BCLC, Barcelona Clinic Liver Cancer; HBV, Hepatitis B Virus; HCV, Hepatitis C Virus; AFP, Alpha-fetoprotein; NLR, Neutrophil to Lymphocyte Ratio; LMR, Lymphocyte to Monocyte Ratio; PLR, Platelet to Lymphocyte Ratio; TACE, Transcatheter Arterial Chemoembolization; c-TACE, conventional TACE; DEB-TACE, Drug Eluting Beads TACE.

Similarly, for overall survival (OS), univariate analysis revealed that BCLC stage, AFP level, NLR, PLR, and TACE response were significant factors. Multivariate analysis confirmed BCLC stage C (HR= 2.355, 95% CI: 1.423–3.898, *P*= 0.001), Child-Pugh class B (HR= 1.665, 95% CI: 1.039–2.669, *P*= 0.034), and an ineffective TACE response (HR= 1.767, 95% CI: 1.113–2.804, *P*= 0.016) as independent predictors of poorer OS. Detailed findings are shown in **Table 3**.

**Table 3:** Uni- and multivariate Cox regression analysis of the risk factors associated with OS.

Variable	Univariate analysis		Multivariate analysis	
	HR (95% CI)	P-value	HR (95% CI)	P-value
Gender		0.343	–	–
Male	Reference			
Female	1.290 (0.762, 2.183)			
Age		0.432		
<60	Reference			
≥60	0.850 (0.567, 1.275)			
BCLC stage		<0.001		0.001
Stage B	Reference		Reference	
Stage C	2.524 (1.623, 3.924)		2.355 (1.423, 3.898)	
HCC lesion rupture		0.550		
Yes	Reference			
No	0.838 (0.474, 1.480)			
Child-Pugh grade		0.070		0.034
A	Reference		Reference	
B	1.514 (0.966, 2.372)		1.665 (1.039, 2.669)	
Hepatitis		0.120		
None	Reference			
HBV	0.835 (0.401, 1.738)			
HCV	0.186 (0.023, 1.487)			
Alcoholic	1.362 (0.587, 3.161)			
AFP (ng/mL)		0.002		0.607
<400	Reference		Reference	
≥400	1.888 (1.256, 2.838)		1.134 (0.704, 1.827)	
NLR	1.060 (1.006, 1.118)	0.030	1.040 (0.948, 1.142)	0.408
LMR	0.881 (0.758, 1.025)	0.101		
PLR	1.002 (1.000, 1.004)	0.039	0.999 (0.996–1.003)	0.740
TACE Type		0.263		
c-TACE	Reference			
DEB-TACE	1.326 (0.809, 2.1.74)			
Initial therapy modality		0.284		
Combination	Reference			
TACE monotherapy	1.277 (0.816, 1.997)			
TACE overall response		0.001		0.016
Effective	Reference		Reference	
Ineffective	2.048 (1.348–3.113)		1.767 (1.113, 2.804)	

OS, Overall Survival; HR, Hazard Ratio; 95% CI, 95% Confidence Interval; BCLC, Barcelona Clinic Liver Cancer; HBV, Hepatitis B Virus; HCV, Hepatitis C Virus; AFP, Alpha-fetoprotein; NLR, Neutrophil to Lymphocyte Ratio; LMR, Lymphocyte to Monocyte Ratio; PLR, Platelet to Lymphocyte Ratio; TACE, Transcatheter Arterial Chemoembolization; c-TACE, conventional TACE; DEB-TACE, Drug Eluting Beads TACE.

### Survival analysis

**Figure 2:**
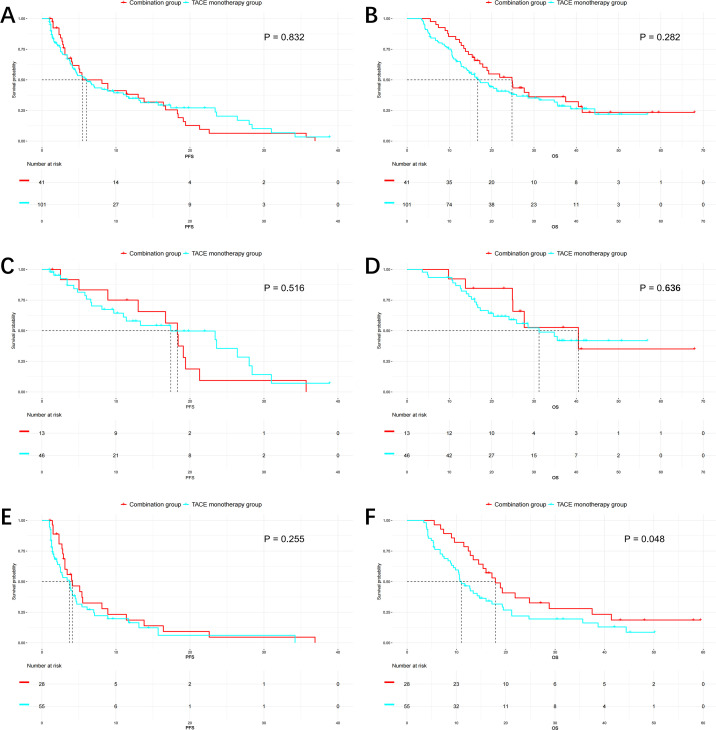
Kaplan–Meier curves comparing progression-free survival (PFS) and overall survival (OS) between TACE monotherapy and TACE combined with systemic therapy. **(A)** PFS in the overall cohort showed no significant difference between groups (median 5.5 vs. 6.0 months, P = 0.832). **(B)** OS in the overall cohort showed a non-significant trend favoring combination therapy (median 24.8 vs. 16.7 months, P = 0.282). **(C)** In BCLC-B patients, PFS was similar between the two groups (median 18.3 vs. 17.4 months, P = 0.516). **(D)** OS in BCLC-B patients showed no significant difference (median 40.5 vs. 31.2 months, P = 0.636). **(E)** In BCLC-C patients, PFS was comparable (median 4.1 vs. 3.7 months, P = 0.255). **(F)** OS was significantly improved in BCLC-C patients receiving combination therapy (median 17.9 vs. 11.0 months, P = 0.048).

Kaplan–Meier survival analysis demonstrated no significant difference in median PFS between the combination therapy group and TACE monotherapy group in the overall cohort (5.5 vs. 6.0 months, P= 0.832, **Figure 2A**). Similarly, in subgroup analysis by BCLC stage, median PFS did not differ significantly between the two treatment groups: 18.3 vs. 17.4 months in BCLC-B patients (P= 0.516, **Figure 2C**), and 4.1 vs. 3.7 months in BCLC-C patients (P= 0.255, **Figure 2E**).

However, the combination therapy group exhibited a trend toward improved OS, particularly in advanced-stage patients. In the overall population, median OS was longer in the combination group compared to the monotherapy group (24.8 vs. 16.7 months), though the difference did not reach statistical significance (P= 0.282, **Figure 2B**). Among BCLC-B patients, median OS was 40.5 vs. 31.2 months (P= 0.636, **Figure 2D**), while in BCLC-C patients, the combination group achieved a significantly longer median OS than the monotherapy group (17.9 vs. 11.0 months, P= 0.048, **Figure 2F**), suggesting a potential survival benefit in advanced HCC patients for TACE combined systemic therapy. A *post-hoc* power analysis yielded a power of 51.3% to detect this difference.

### Post-TACE adverse events

No treatment-related death occurred during the initial TACE procedure. Grade 3 or 4 treatment-related AEs were observed in 3 patients (7.2%) in the combination group and 15 patients (14.9%) in the monotherapy group (P= 0.187). The most commonly reported events included high fever, nausea, severe abdominal pain, and liver function damage. No statistically significant differences were observed between groups for any individual AE. Notably, serious complications such as liver abscess, cardiac injury, or HCC lesion rupture were rare and occurred only in the monotherapy group. The detailed date is presented in **Table 4**.

**Table 4:** Post TACE adverse events.

AEs	CTCAE grade	Combination group (n=41) (%)	TACE monotherapy group (n=101) (%)	*P* value
Fever (> 40°C)	3	2 (4.9)	4 (4.0)	0.805
Severe abdominal Pain	3	0 (0)	4 (4.0)	0.196
Nausea	3	1 (2.4)	5 (5.0)	0.502
Pleural effusion or ascites	3	0 (0)	3 (3.0)	0.265
Liver abscess	4	0 (0)	1 (1)	0.523
HCC lesion rupture	4	0 (0)	2 (2.0)	0.364
Cardiac function damage	3	0 (0)	3 (3.0)	0.265
Liver function damage	3	0 (0)	2 (2.0)	0.364
Severe diarrhea	3	0 (0)	2 (2.0)	0.364
Total grades 3/4 AEs	–	3 (7.2)	15 (14.9)	0.187

TACE, Transcatheter Arterial Chemoembolization; Aes, Adverse Events; CTCAE, Common Terminology Criteria for Adverse Events.

## Discussion

This retrospective study evaluated the efficacy of combining systemic therapy with TACE compared to TACE monotherapy in patients with intermediate and advanced stage HCC. Our findings suggest that while combination therapy does not significantly improve PFS across the overall cohort or within BCLC B and C subgroups, it is associated with a trend toward improved OS, particularly in patients with BCLC stage C disease. The statistically significant OS benefit observed in BCLC-C patients (17.9 vs. 11.0 months, P= 0.048) highlights the potential of systemic therapy to augment TACE in advanced-stage HCC, despite the lack of PFS improvement.

The absence of a significant PFS benefit in the combination group aligns with findings from prior trials, such as SPACE and TACE-2, which reported no significant survival advantage when combining TACE with sorafenib (5, 6). Several factors may explain this observation. First, the heterogeneity of BCLC B patients, encompassing variations in tumor burden, AFP levels, and liver function, likely influences treatment response (7, 19). Our multivariate analysis identified BCLC stage C and ineffective TACE response as independent predictors of shorter PFS, suggesting that tumor biology and initial treatment response are critical determinants of disease progression (20). Second, the retrospective nature of our study and the relatively small sample size (n=142) may limit the statistical power to detect subtle differences in PFS, particularly in the BCLC-B subgroup, where median PFS was comparable between groups (18.3 vs. 17.4 months, P= 0.516).

In contrast, the trend toward improved OS in the combination group, particularly in BCLC-C patients, is consistent with results from trials like CHANCE001 and CHANCE2211, which reported OS benefits of 19.2–24.1 months with TACE plus targeted-immunotherapy (1, 2). The significant OS improvement in BCLC-C patients (17.9 vs. 11.0 months, P= 0.048) may reflect the synergistic effects of systemic therapies, such as immune checkpoint inhibitors or targeted agents, in controlling systemic disease spread and microvascular invasion, which are more prevalent in advanced HCC (21, 22). For the OS benefit observed in BCLC-C patients, a *post-hoc* power analysis yielded a value of 51.3%. Although the statistical power was constrained by the sample size, the attainment of statistical significance (P= 0.048) highlights the potent synergistic effect of TACE combined with systemic therapy in this high-risk population. Systemic therapies like atezolizumab plus bevacizumab have shown efficacy in high-risk HCC populations, including those with portal vein tumor thrombosis (PVTT), and our findings suggest that combining such therapies with TACE may enhance tumor control in similar high-risk cohorts (23).

From a mechanistic perspective, this PFS-OS mismatch may be explained by several complementary mechanisms. TACE-induced ischemia and necrosis can trigger immunogenic cell death (ICD), resulting in the systemic release of damage-associated molecular patterns (DAMPs) and tumor-associated antigens (TAAs) (24). This process promotes T-cell recruitment and activation, thereby priming a durable anti-tumor immune response that may suppress occult micro-metastases and ultimately translate into improved overall survival rather than an immediate progression-free survival benefit (25). In BCLC-C patients, whose disease is often characterized by vascular invasion and systemic dissemination (e.g., PVTT), the addition of vascular endothelial growth factor receptor (VEGFR) inhibitors and PD-1/PD-L1 inhibitors complements the local effect of TACE by targeting angiogenesis and immune evasion, helping control extrahepatic and microvascular spread (7). Moreover, the higher proportion of Child–Pugh A patients in the combination group likely enabled better tolerance of therapy and greater access to subsequent treatments after progression, further contributing to the observed survival advantage (1, 5).

The safety profile of combination therapy was comparable to TACE monotherapy, with no significant increase in grade 3 or 4 adverse events (AEs) (7.2% vs. 14.9%, P= 0.187). This contrasts with concerns raised in prior studies, such as SPACE and TACE-2, which reported increased severe AEs, including liver dysfunction and immune-related hepatitis, with TACE plus sorafenib (5, 6). The lower AE rate in our combination group may be attributed to the higher proportion of Child-Pugh A patients (90.2% vs. 67.3%, P= 0.005), indicating better baseline liver function and potentially greater tolerance to combined regimens. This finding underscores the importance of patient selection, as those with preserved liver function (Child-Pugh A) may better tolerate the cumulative toxicity of combination therapy. In contrast, Child-Pugh B patients, who had poorer OS in our multivariate analysis (HR= 1.665, P= 0.034), may require cautious consideration (26).

Patient selection remains a critical challenge in optimizing TACE plus systemic therapy (27). Our study suggests that BCLC-C patients, who often present with high-risk features like elevated AFP or PVTT, may derive greater OS benefit from combination therapy. Conversely, BCLC-B patients with smaller tumor burdens may achieve adequate control with TACE alone, as evidenced by the comparable PFS and OS in this subgroup. These findings align with the literature, which indicates that HCC patients with lower tumor burden may not require systemic therapy, while high-risk patients benefit from its addition (28). Inflammatory biomarkers (e.g., NLR, PLR) were significant predictors of PFS and OS in univariate analyses, consistent with their established role as prognostic indicators in HCC (15, 29). However, their lack of significance in multivariate models suggests that tumor stage and treatment response may outweigh inflammatory indices in determining outcomes.

The cost-effectiveness of TACE combined with systemic therapy remains a concern, particularly in resource-constrained settings (30). While our study did not assess economic outcomes, the high cost of systemic therapies, such as immune checkpoint inhibitors, may limit their accessibility. Future studies should incorporate cost-effectiveness analyses to guide resource allocation and treatment decision-making, especially in regions with limited healthcare budgets.

Our study has several limitations. First, its retrospective design introduces potential selection bias, as treatment allocation was not randomized. Second, the sample size, particularly in the combination group (n=41), may limit the detection of statistically significant differences in PFS. Third, the specific systemic therapies regimen were not standardized, which may influence outcomes due to differences in mechanisms of action. Then, A key limitation is the baseline imbalance in liver function, with more Child–Pugh A patients in the combination group (90.2% vs 67.3%, P= 0.005), which may bias OS. However, given the small sample size, especially in the BCLC-C combination subgroup (n=28), we did not perform PSM/IPTW to avoid loss of statistical power and instead adjusted for Child–Pugh status in multivariate Cox regression, warranting validation in larger multicenter cohorts. Finally, the single-center setting may limit the generalizability of our findings, necessitating validation in larger, multicenter cohorts.

## Conclusion

In conclusion, our study suggests that combining systemic therapy with TACE does not significantly improve PFS in intermediate-stage HCC but may offer an OS benefit, particularly in BCLC-C patients. The comparable safety profile of combination therapy, especially in patients with preserved liver function, supports its potential in high-risk populations. However, patient selection, tumor characteristics, and cost considerations remain critical for optimizing treatment strategies. Future prospective trials are needed to refine indications for TACE plus systemic therapy and to establish its cost-effectiveness in diverse healthcare settings.

## Data Availability

The raw data supporting the conclusions of this article will be made available by the authors, without undue reservation.
